# Death of the President of the United States

**Published:** 1850-08

**Authors:** 


DEATH OF THE PRESIDENT OF THE UNITED STATES.

This melancholy event, which has shrouded our land in mourning, took place under circumstances which are well calculated to illustrate the importance of a much stricter regard to the laws of health and life, of which we have taken occasion to speak in a former number of the *Gazette*, than is usually exercised on the part of aged persons, whose vitality is inadequate to sustain a severe shock of disease.

It has been stated in the newspapers, that the death of Gen. Taylor was caused by a bilious remittent fever of congestive character, superinduced by an attack of Cholera Morbus. But what were the pre-existing causes to which the onset of this latter disease is to be ascribed? On the same authority we learn that the illustrious chief passed the day in a crowded assembly participating in its celebration by exposure to a temperature of near 90° in the shade, until he returned home in a state of exhaustion and hunger, which prompted him to indulgence of a full meal, of which he felt urgent need. Of the extent of this meal, and the variety of dishes of which he partook, we have not been accurately informed, but it is stated that he ate heartily of *boiled cabbage, stringed beans, cucumbers, cherries and other raw fruit, with milk,* while his system had been enfeebled by long fasting, toil and heat, as well as by the excitement incident to the occasion.

That such a meal, under such circumstances, by a man of 66 years of age, was indiscreet, cannot admit of a doubt, even if these several articles were eaten in moderation as to quantity. Digestion and nutrition of such a medley of ingesta, were wholly out of the question, and hence all these combustibles became subject to chemical laws; and fermentation in the stomach, and the subsequent explosion called Cholera Morbus, might have been predicted with certainty, especially in a temperate man, who habitually abstained from stimulating drinks, as did General Taylor.—*N. Y. Med. Gaz.*

